# Cilostazol Modulates Autophagic Degradation of β-Amyloid Peptide via SIRT1-Coupled LKB1/AMPKα Signaling in Neuronal Cells

**DOI:** 10.1371/journal.pone.0160620

**Published:** 2016-08-05

**Authors:** So Youn Park, Hye Rin Lee, Won Suk Lee, Hwa Kyoung Shin, Hye Young Kim, Ki Whan Hong, Chi Dae Kim

**Affiliations:** 1 Department of Pharmacology, School of Korean Medicine, Pusan National University, Gyeongsangnam-do, 50612, Republic of Korea; 2 Gene & Cell Therapy Research Center for Vessel-associated Diseases, Pusan National University, Gyeongsangnam-do, 50612, Republic of Korea; 3 Division of Meridian and Structural Medicine, Pusan National University, Gyeongsangnam-do, 50612, Republic of Korea; IISER-TVM, INDIA

## Abstract

A neuroprotective role of autophagy mediates the degradation of β-amyloid peptide (Aβ) in Alzheimer’s disease (AD). The previous study showed cilostazol modulates autophagy by increasing beclin1, Atg5 and LC3-II expressions, and depletes intracellular Aβ accumulation. This study elucidated the mechanisms through which cilostazol modulates the autophagic degradation of Aβ in neurons. In N2a cells, cilostazol (10–30 μM), significantly increased the expression of P-AMPKα (Thr 172) and downstream P-ACC (acetyl-CoA carboxylase) (Ser 79) as did resveratrol (SIRT1 activator), or AICAR (AMPK activator), which were blocked by KT5720, compound C (AMPK inhibitor), or sirtinol. Furthermore, phosphorylated-mTOR (Ser 2448) and phosphorylated-P70S6K (Thr 389) expressions were suppressed, and LC3-II levels were elevated in association with decreased P62/Sqstm1 by cilostazol. Cilostazol increased cathepsin B activity and decreased p62/SQSTM 1, consequently decreased accumulation of Aβ1–42 in the activated N2aSwe cells, and these results were blocked by sirtinol, compound C and bafilomycin A1 (autophagosome blocker), suggesting enhanced autophagosome formation by cilostazol. In SIRT1 gene-silenced N2a cells, cilostazol failed to increase the expressions of P-LKB1 (Ser 428) and P-AMPKα, which contrasted with its effect in negative control cells transfected with scrambled siRNA duplex. Further, N2a cells transfected with expression vectors encoding pcDNA SIRT1 showed increased P-AMPKα expression, which mimicked the effect of cilostazol in N2a cells; suggesting cilostazol-stimulated expressions of P-LKB1 and P-AMPKα were SIRT1-dependent. Unlike their effects in N2a cells, in HeLa cells, which lack LKB1, cilostazol and resveratrol did not elevate SIRT1 or P-AMPKα expression, indicating cilostazol and resveratrol-stimulated expressions of SIRT1 and P-AMPKα are LKB1-dependent. In conclusion, cilostazol upregulates autophagy by activating SIRT1-coupled P-LKB1/P-AMPKα and inhibiting mTOR activation, thereby decreasing Aβ accumulation.

## Introduction

Alzheimer’s disease (AD) is characterized by extracellular β-amyloid (Aβ)-containing plaques and intracellular neurofibrillary tangles (NFT) that result in synaptic and neuronal failure and cognitive deficits [1]. Theoretically, Aβ accumulation can be reduced in AD patients by suppressing its production or by enhancing its degradation and/or clearance [2]. Autophagy plays an active role in healthy neurons, and protects them from Aβ-induced cytotoxicity [3–7]. Thus, autophagy provides potential means of decreasing Aβ aggregates in neurons and alleviating neurotoxicity. Many studies have documented that macroautophagy is impaired in the AD brain, and that as a result Aβ-containing autophagic vacuoles accumulate and enhance neurodegenerative pathology [3,5].

AMP-activated protein kinase (AMPK, a heterotrimeric serine/threonin protein kinase) is an emerging key regulator of whole-body metabolism, and has been shown to increase NAD^+^ levels and activate SIRT1 and PGC-1 [8,9]. In addition, Aβ is known to increase mTOR (mammalian target of rapamycin) signaling, and decreasing of the mTOR is known to reduce Aβ levels, which suggest an interrelationship between mTOR signaling and Aβ [6]. It has also been reported AMPK activation is required for autophagy by repressing mTOR, a key blocker of autophagosome formation [10,11], and that AMPK activator potently inhibits mTOR signaling and then promotes autophagy and triggers Aβ degradation by the lysosomal system [12].

SIRT1 that is induced by calorie restriction in tissues is importantly involved in metabolic changes [13]. Many studies have shown calorie restriction prevents AD-type amyloid neuropathology in animal models [14,15], and Qin et al. [16] proposed SIRT1 activation in brain by calorie restriction modulates amyloid neuropathology in the AD brain. Furthermore, it is has also been reported that the mTORC1 pathway is regulated by SIRT1 and AMPK [17].

Cilostazol is known to increase intracellular cyclic AMP (cAMP) levels by inhibiting type III phosphodiesterase [18]. Cilostazol has been demonstrated to reduce the cortical infarct size by increasing cyclic AMP levels [19]. A pilot study was also reported on 10 patients with moderate Alzheimer’s disease in a clinical setting where combination therapy with cilostazol and donepezil significantly improved the Mini-Mental State Exam (MMSE) score and maintained the current status unchanged until the end of the follow-up period in human patients with AD [20]. Most recently, Ihara et al. [21] have noted a potential for cilostazol treatment in the preservation of cognitive function in patients with early-stage cognitive impairment. Cilostazol was recently reported to cause significant reductions in intracellular Aβ accumulation and to decrease phosphorylated tau content in N2aSwe cells (N2a cells stably expressing human APP Swedish mutation), and to improve spatial learning and memory in C57BL/6J mice administered an intracerebroventricular injection of Aβ_25–35_ [22].

In the previous reports, Lee et al. [23] reported a time-dependent decrease (3, 12, 24 hr) in SIRT1 protein expression in the activated N2aSwe cells containing endogenously overproduced Aβ, and the decreased SIRT1 expression was elevated by cilostazol (3–10 μM) as did resveratrol (20 μM). In addition, it was demonstrated cilostazol-stimulated SIRT1 activation suppressed tau acetylation and phosphorylation by inhibiting the activations of P300 and GSK3β, and decreasing Aβ expression in N2aSwe cells [23].

Further, they showed cilostazol modulates autophagy machinery by increasing SIRT1 activation and beclin-1, Atg5, and LC3-II expressions, thereby results in depletion of intracellular Aβ and of C-terminal APP fragment β subunit (APP-CTF) [24]. Nevertheless, it remains unknown as to the mechanism (s) by which cilostazol leads to the autophagic degradation of Aβ in neurons.

Given (1) autophagy is a major cellular pathway leading to the degradation of intracellular Aβ, and (2) cilostazol enhances autophagy by increasing SIRT1 expression and activity, we hypothesized that AMPK activation by cilostazol may enhance autophagy by suppressing mTOR, and thereby increase the autophagic clearance of Aβ. Based on these hypotheses, we examined that cilostazol enhances the phosphorylation of AMPKα at Thr172, and subsequently inhibits the activity of mTOR (an AMPK target involved in autophagy repression), and compared the results with those of the AMPK activator AICAR. Herein, we show cilostazol upregulates autophagy through activating SIRT1-coupled P-LKB1/P-AMPKα and inhibiting mTOR activation, thereby decreasing Aβ accumulation.

## Results

### Activation of P-AMPKα and P-ACC by cilostazol in N2a Cells

**Fig 1:**
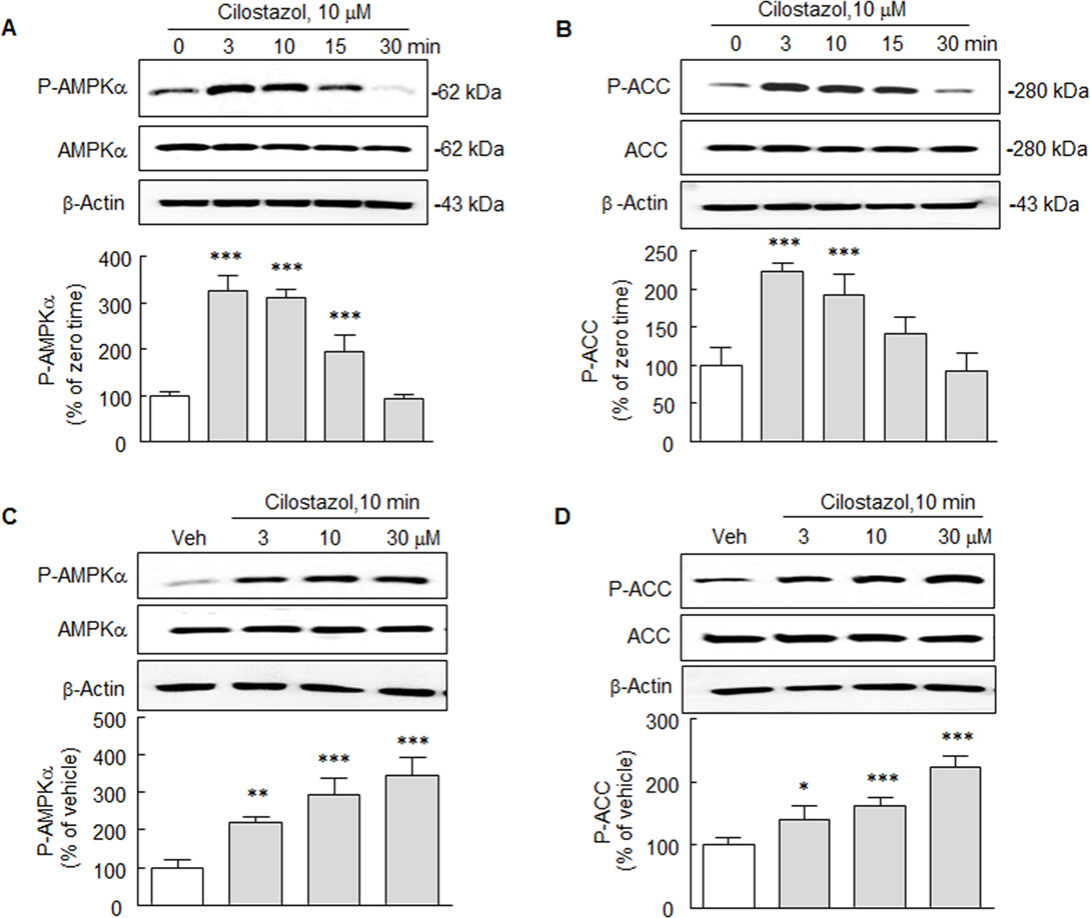
**A** and **B**. Time-course effect of cilostazol. Cilostazol (10 μM) stimulated increases in P-AMPKα and P-ACC expressions, both of which peaked at 3 min and then declined. Total AMPKα and ACC levels were unchanged. **C** and **D**. Cilostazol (3, 10, or 30 μM) concentration-dependently increased the expressions of P-AMPKα and P-ACC. Total AMPKα and ACC levels remained unchanged. Means ± SDs are expressed as percentages of zero time or vehicle (Veh) values (N = 4 ~ 5). *P < 0.05, **P < 0.01, ***P < 0.001 vs. Zero time or Veh.

Under treating N2a cells with cilostazol, time (3, 10, 15 and 30 min)-dependent changes in AMPKα phosphorylation at Thr 172 (P-AMPKα) and its downstream target acetyl-CoA carboxylase phosphorylation at Ser 79 (P-ACC) were determined by Western blot. After treating for 3–10 min with cilostazol (10 μM), P-AMPKα levels increased by 327.5 ± 31.7% (*P* < 0.001) and then slowly declined (*F*_4,20_ = 118.1, *P* < 0.0001) to the basal level at 30 min. Similarly, levels of P-ACC, a primary target of activated AMPK, increased to 223.3 ± 10.6% at 3 min and then decreased to baseline at 30 min (*F*_4,20_ = 25.6, *P* < 0.0001) (Fig 1A and 1B). Furthermore, treatment with cilostazol (3, 10 and 30 μM) for 10 min significantly and concentration-dependently increased P-AMPKα (*F*_3,12_ = 37.9, *P* < 0.0001) and P-ACC (*F*_3,12_ = 39.5, *P* < 0.0001) expressions. Nevertheless, both AMPKα and ACC levels were little changed by cilostazol treatment (Fig 1C and 1D).

### Comparison of cilostazol with resveratrol and AICAR

**Fig 2:**
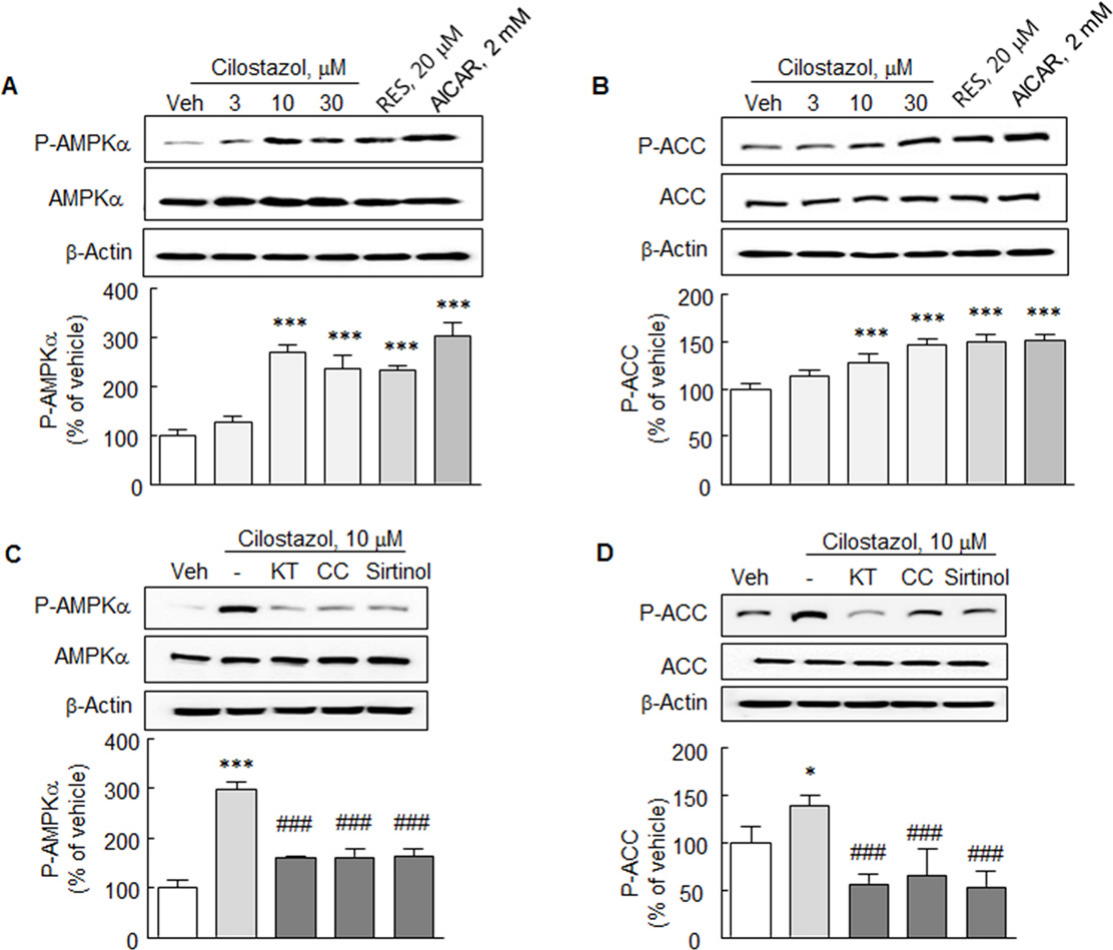
**A** and **B**. Comparison of the effects of cilostazol (3, 10 or 30 μM), resveratrol (RES, 20 μM), and AICAR (2 mM) on the expressions of P-AMPKα and P-ACC. **C** & **D**. Inhibition of cilostazol (10 μM)-stimulated expressions of P-AMPKα and P-ACC by KT5720 (KT, 10 μM; cAMP-dependent protein kinase inhibitor), compound C (CC, 10 μM; inhibitor of AMPK), or sirtinol (20 μM; inhibitor of SIRT1). Means ± SDs are expressed as percentages of vehicle (Veh) values (N = 4). *P < 0.05, ***P < 0.001 vs. Veh; ^###^*P* < 0.001 vs. cilostazol (10 μM) alone.

The effects of cilostazol on P-AMPKα and its downstream target P-ACC were compared figwith those of resveratrol (SIRT1 activator) and AICAR (AMPK activator) in N2a cells. As shown in Fig 2A and 2B, cilostazol (10–30 μM) significantly increased both P-AMPKα and P-ACC in a concentration-dependent manner as did resveratrol (20 μM) and AICAR (2 mM). These observations suggested a close relation between cilostazol and the activations of SIRT1 and AMPK.

This suggestion was further identified by examining whether increases in P-AMPKα and P-ACC by cilostazol were inhibited by KT5720 (cAMP-dependent protein kinase inhibitor, 10 μM), compound C (a chemical inhibitor of AMPK, 50 μM) and sirtinol (a SIRT1 inhibitor, 20 μM). As shown in Fig 2C and 2D, cilostazol (10 μM)-stimulated increases in P-AMPKα and P-ACC were significantly blocked by KT5720 (10 μM), compound C (10 μM), and by sirtinol (20 μM). These results support the notion that cilostazol exerts its effect by activating SIRT1 and AMPK.

### Inhibition of phosphorylation of mTOR and of its downstream P70S6K

**Fig 3:**
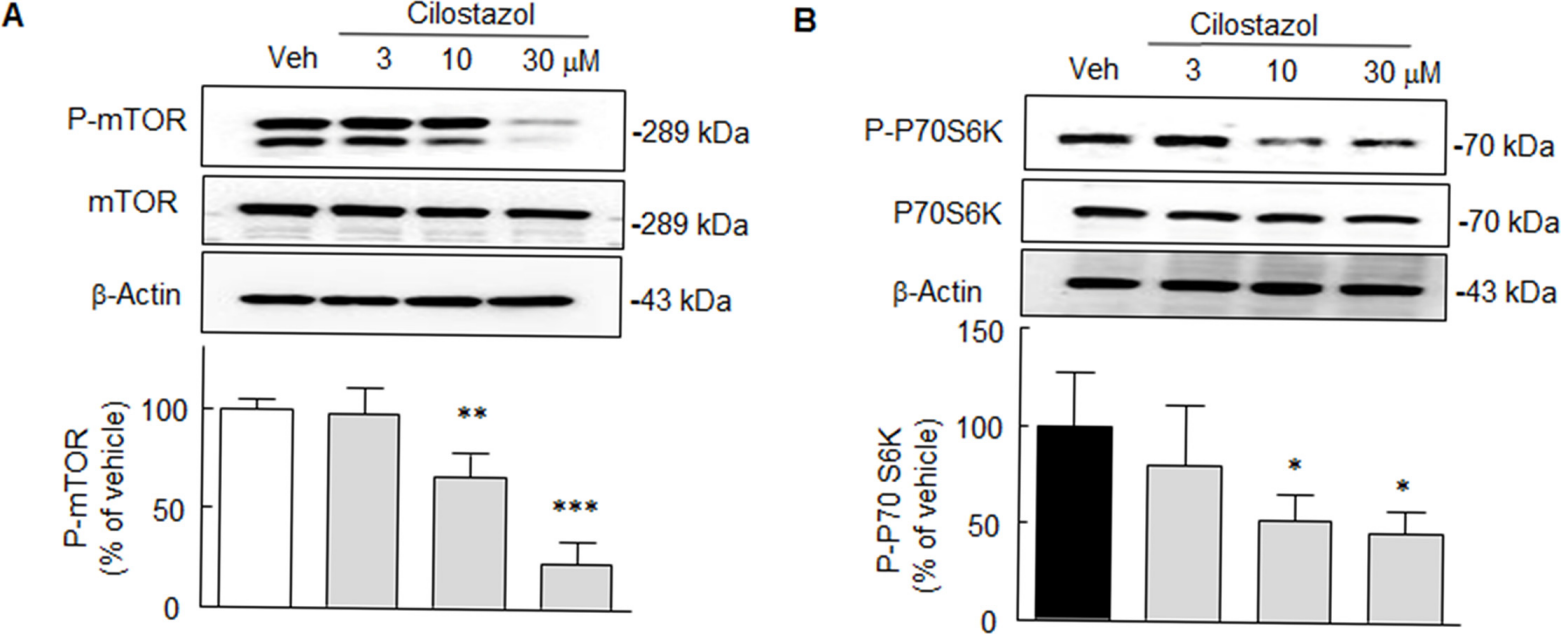
Downregulations of P-mTOR (**A**) and P-P70S6K (**B**) by cilostazol (10 or 30 μM) in N2a cells; total mTOR and P70S6K levels remained unchanged. Means ± SDs are expressed as percentages of vehicle (Veh) (N = 4). *P < 0.05, **P < 0.01, ***P < 0.001 vs. Veh.

Cell extracts from N2a cells were then analyzed by Western blot for mTOR phosphorylation at Ser 2448 (P-mTOR) and P70S6K phosphorylation at Thr 389 (P-P70S6K) expression. mTOR is an inhibitor of macroautophagy, a conserved intracellular system designed to degrade long-lived proteins and organelles in lysosomes [6]. In the present study, P-mTOR expression was significantly decreased by cilostazol at 10–30 μM (*F*_3,12_ = 28.85, *P* < 0.0001). In line with these results, P-P70S6K was also significantly decreased by cilostazol at 10 and 30 μM to 52.2 ± 13.5% and 46.4 ± 11.1%, respectively (*F*_3,12_ = 4.95, *P* < 0.02) (Fig 3A and 3B).

### Expression of LC3-II, P62/Sqstm1 and Cathepsin B activation

**Fig 4:**
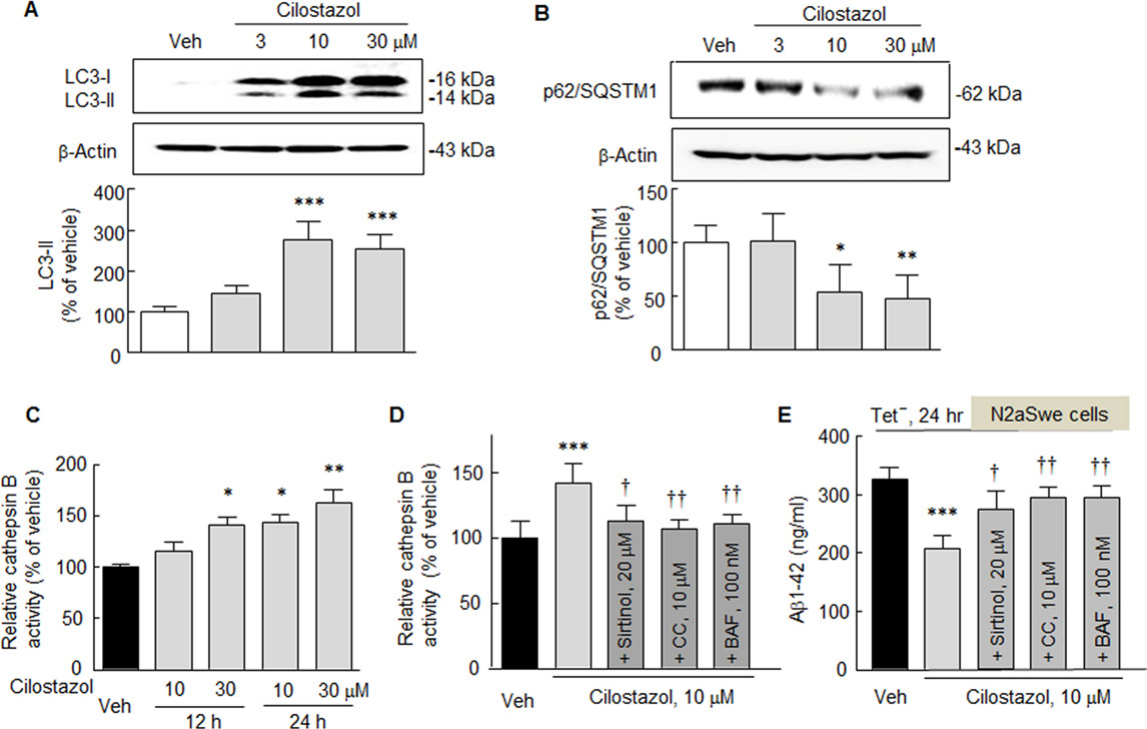
**A**. Significant enhancement of LC3-II levels by cilostazol (10 or 30 μM). **B**. Concentration-dependent decrease in p62/SQSTM 1 expression by cilostazol (10 and 30 μM. **C**. Confirmation of cilostazol-stimulated lysosomal activation in the lysates of N2a cells. Relative cathepsin B activity was significantly increased by cilostazol in time- and concentration-dependent manner. **D**. Blockade of cilostazol-induced cathepsin B activity by sirtinol (20 μM), compound C (CC, 10 μM), or bafilomycin A1 (BFA, 100 nM). **E**. Cilostazol-suppression of Aβ1–42 production which was induced by the Tet^-^ condition (24 hr) in N2aSwe cells, and the blockade of cilostazol inhibition by sirtinol, compound C and bafilomycin A1. Means ± SDs are expressed as percentages of vehicle (Veh) (N = 4). *P < 0.05, **P < 0.01, ***P < 0.001 vs. Veh. ^†^ *P* < 0.05, ^††^*P* < 0.01 vs. cilostazol (10 M) alone.

The effect of cilostazol was further examined with respect to autophagy by determining LC3-II (light chain 3, a marker for autophagy induction) expression in N2a cells treated with or without cilostazol (10 or 30 μM). As shown in Fig 4A, LC3-II levels in N2a cells were significantly elevated by 278 ± 42.8% and 254.6 ± 34.4% by cilostazol at 10 and 30 μM, respectively (*F*_3,12_ = 33.70, *P* < 0001), indicating that cilostazol enhanced autophagosome formation.

It is known p62/SQSTM 1 (p62, a selective substrate for autophagy) is degraded by autophagy through direct interaction with LC3 [25]. Thus, we clarified whether p62 is degraded by cilostazol through autophagy-lysosome pathway. As shown in Fig 4B, p62 expression was significantly decreased by cilostazol at 10 and 30 μM to 54.4 ± 24.9% (*P* < 0.05) and 48,4 ± 21.4% (*P* < 0.01), respectively. Further, we confirmed cilostazol-stimulated lysosomal (cathepsin B) activation in the lysates of N2a cells. Relative cathepsin B activity was significantly increased by cilostazol in a time- and concentration-dependent manner. Interestingly, cilostazol-induced increased cathepsin B activation was prevented by sirtinol, compound C, or bafilomycin A1 (100 nM, a blocker of autophagosome to lysosome fusion [26] (Fig 4C and 4D). These results suggest that increased expression of LC3-II and lysosomal activation by cilostazol lead to increased p62 degradation.

We further verified whether suppression of endogenous Aβ accumulations by cilostazol were blocked by sirtinol, compound C, or bafilomycin A1 (100 nM). For this experiment, mouse neuroblastoma cells stably expressing human APP Swedish mutation (N2aSwe cells) were exposed to Tet^+^ or Tet^-^ conditions as described by Anekonda et al. [27]. Briefly, cells were exposed to medium containing 1 μg/ml of tetracycline (Tet^+^) for 48 hr, and then removed to tetracycline-free (Tet^-^) conditions for 24 hr to induce endogenous Aβ overproduction. When N2aSwe cells were exposed to Tet^-^ medium, intracellular Aβ1–42 levels, as determined by ELISA, significantly increased by 327.0 ± 18.6 ng/ml, but pretreatment with cilostazol (10 μM) markedly reduced to 207.9 ± 21.8 ng/ml (*P* < 0.001). Furthermore, this cilostazol inhibition was significantly blocked by pretreating with sirtinol (20 μM, *P* < 0.05), compound C (10 μM, *P* < 0.01), or bafilomycin A (100 nM, *P* < 0.01), respectively (Fig 4E). These results indicate that cilostazol reduces Aβ accumulation by upregulating autophagy by increasing lysosome activity through activations of SIRT1 and P-AMPKα.

### SIRT1 gene knockdown and Overproduction

**Fig 5:**
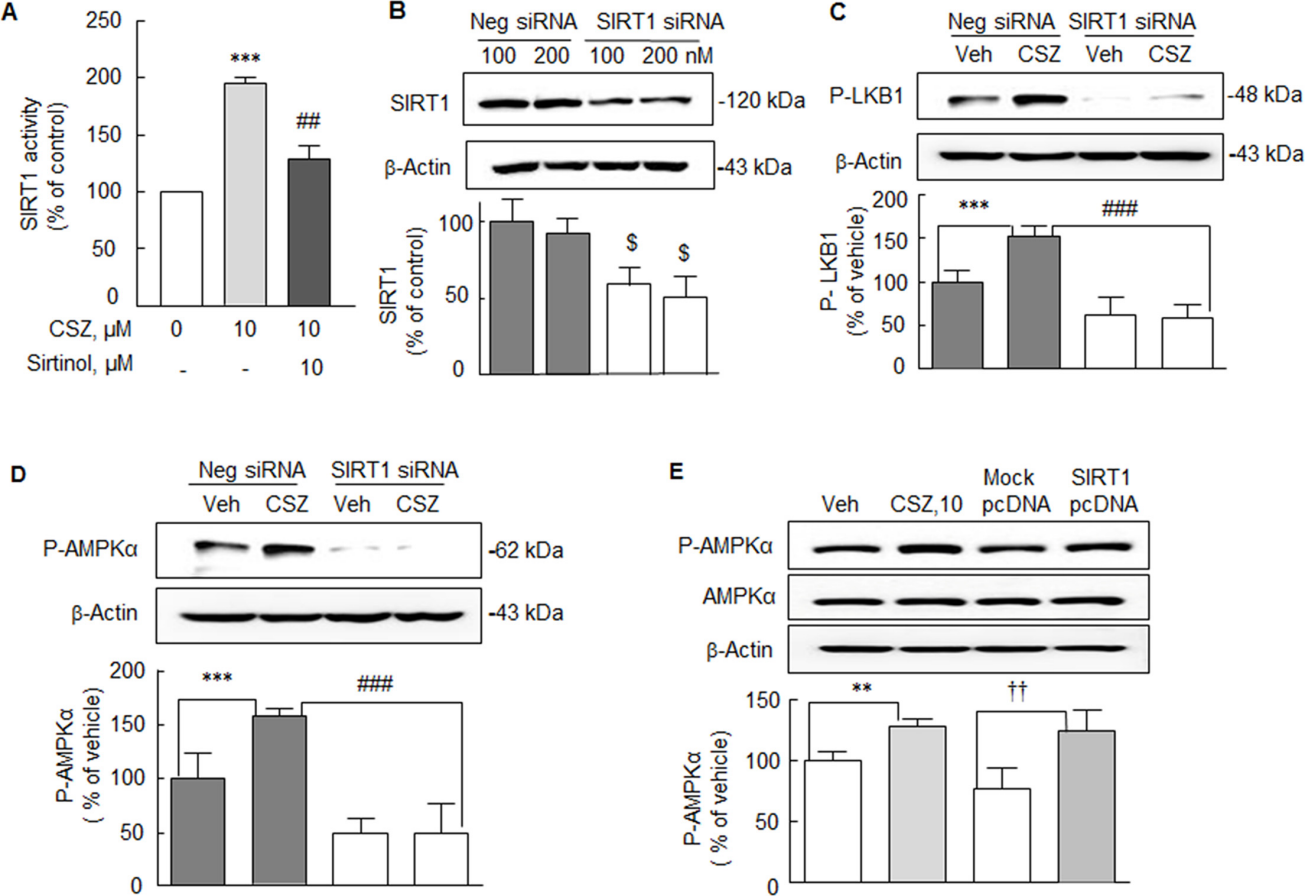
**A.** Stimulation of SIRT1 deacetylase activity by cilostazol (CSZ, 10 μM) in the N2a cells and blockade by sirtinol (10 μM, *P* < 0.01). Means ± SDs are expressed as percentages of none (N = 4). ***P < 0.001 vs. none; ^##^*P* < 0.01 vs. cilostazol (10 μM) alone. **B.** Analysis of the effects of SIRT1-knockdown in N2a cells. In N2a cells transfected with 200 nM of SIRT1 siRNA, SIRT1 protein levels were reduced to ~ 40% of the level in negative controls. Cilostazol (10 μM) failed to elevate the expressions of P-LKB1 (**C**) and P-AMPKα (**D**) in SIRT1 siRNA-transfected N2a cells, which contrasted with its effects in negative controls. Means ± SDs are expressed as percentages of vehicle in negative siRNA (N = 4). ^$^*P* < 0.05 vs. Negative (Neg) siRNA; ***P < 0.001 vs. Veh; ^###^*P* < 0.001 vs. cilostazol effect of negative siRNA. **E**. N2a cells were transfected with expression vectors encoding pcDNA SIRT1 or mock pcDNA. Increased P-AMPKα expression by cilostazol (CSZ, 10 μM) in N2a cells was compared with that induced by SIRT1 gene-overexpressing cells in the absence of cilostazol. Significantly increased P-AMPKα expression in SIRT1 pcDNA-transfected cells was evident in the absence of cilostazol. Means ± SDs are expressed as percentages of vehicle (Veh) values (N = 4). **P < 0.01 vs. Veh. ^††^*P* < 0.01 vs Mock pcDNA.

Consistent with the previous report [23], cilostazol (10 μM) significantly stimulated SIRT1 deacetylase activity in the N2a cells to 195.5 ± 9.7% (*P* < 0.001), and this increase was attenuated by pretreatment with sirtinol (10 μM) to 129.7 ± 11.6% (*P* < 0.01) (Fig 5A).

To confirm whether cilostazol-stimulation of LKB1 phosphorylation at Ser 428 (P-LKB1) and of P-AMPKα, downstream of P-LKB1, are mediated via SIRT1 activation, N2a cells were transfected with SIRT1 siRNA (100 nM). In N2a cells transfected with 200 nM of SIRT1 siRNA, SIRT1 protein levels were reduced to ~ 40% of the level in negative controls (Fig 5B). In the N2a cells subjected to SIRT1-knockdown, cilostazol failed to increase the expressions of P-LKB1 or P-AMPKα while it increased their expressions in negative control cells transfected with scrambled siRNA duplex (Fig 5C and 5D).

Further, increased P-AMPKα expression by cilostazol (10 μM) in the wild type N2a cells was compared with that in SIRT1 gene-overexpressing cells in the absence of cilostazol. When N2a cells were transfected with expression vectors encoding pcDNA SIRT1 or mock pcDNA, the increase in P-AMPKα expression in cells transfected with pcDNA SIRT1 was similar to that induced by cilostazol, whereas P-AMPKα expression was not increased in mock pcDNA-transfected cells (Fig 5E). Overall, these results indicate that the cilostazol stimulated expressions of P-LKB1 and P-AMPKα are SIRT1-dependent.

### Immunofluorescence studies

**Fig 6:**
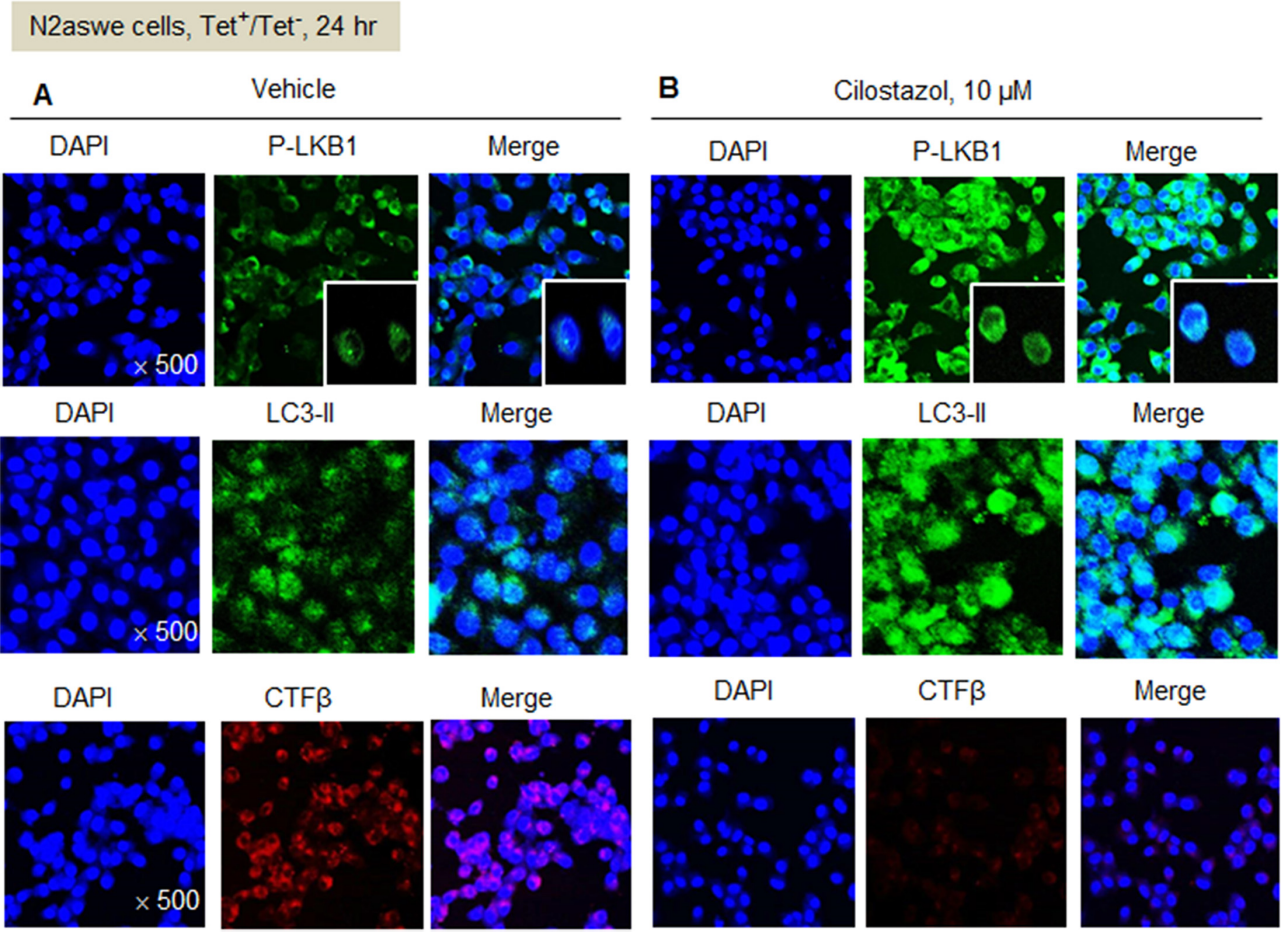
Immunofluorescence studies: Representative photographs showing excessive P-LKB1/LC3-II (+) expression and markedly diminished CTFβ(+)-cells under treatment with cilostazol (10 μM) in the activated N2aSwe cells, as compared with vehicle-treated group.

An immunofluorescence approach was adopted to assess the relationships between CTFβ and P-LKB1/LC3-II in activated N2aSwe cells which were pretreated with vehicle and cilostazol. Cells pretreated with or without cilostazol (10 μM) were exposed to medium containing 1 μg/ml of tetracycline (Tet^+^) for 48 h and then exposed to Tet^-^ for 24 hr to induce endogenous CTFβ overproduction. For vehicle-treated N2aSwe cells, P-LKB1 and LC3-II-positive cells were markedly diminished and CTFβ-positive cells were markedly increased, whereas for cilostazol-pretreated cells, P-LKB1 and LC3-II positive cells were prominently increased and in contrast CTFβ positive cells were almost abolished (Fig 6A and 6B). Overall, these results confirmatively indicate that cilostazol stimulates the conversion of LC3-I to LC3-II via P-LKB1, and thereby reduces CTFβ accumulation.

### Functional relevance of P-LKB1 to P-AMPK signaling

**Fig 7:**
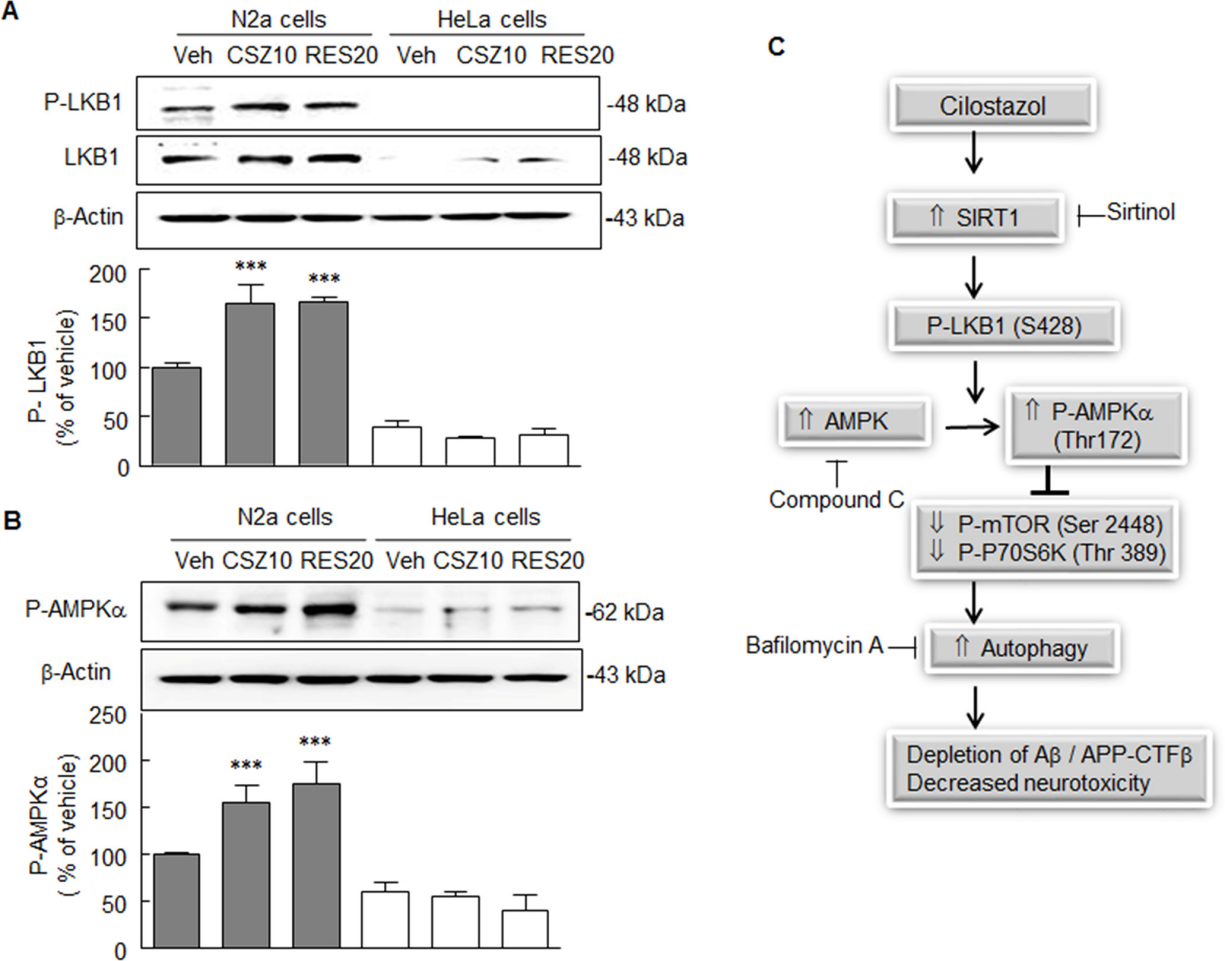
Comparison of cilostazol- and resveratrol-stimulated P-LKB1 and P-AMPKα expressions in N2a cells that LKB1 is detectable, and in HeLa cells that lack LKB1. **A**. Immunoblot of P-LKB significantly increased after pretreating N2a cells with cilostazol (CSZ, 10 μM) or resveratrol (RES, 20 μM), whereas P-LKB expression was not appeared in HeLa cells. **B**. The significant increases in P-AMPK expression by cilostazol (CSZ, 10 μM) or resveratrol (Res, 20 μM) were not observed in HeLa cells, whereas they were obviously identified in N2a cells. Results are expressed as the means ± SDs of 4 experiments. ***P < 0.001 vs. vehicle (Veh). **C**. Proposed signal pathways for the neuroprotective effect of cilostazol against Aβ-induced neurotoxicity: Cilostazol upregulates autophagy through activating SIRT1/LKB1/AMPK1α signal pathways and depletes intracellular Aβ and APP-CTFβ accumulation, and thereby results in decreased neurotoxicity.

The expressions of cilostazol- or resveratrol-stimulated P-LKB1, SIRT1, and P-AMPKα were compared between N2a cells that express LKB1 and HeLa cells that lack LKB1, representing a natural “knockout” cell line [28]. In HeLa cells treated with or without cilostazol or resveratrol, P-LKB1 and LKB1 were not expressed, but in N2a cells, P-LKB1 expression was significantly increased by cilostazol (10 μM, *P* < 0.001) and resveratrol (20 μM, *P* < 0.001) (Fig 7A). Similarly to these results, P-AMPKα expression was not induced by either cilostazol or resveratrol in HeLa cells, whereas P-AMPKα expression was significantly elevated to 156.4 ± 17.9 (*P* < 0.001) by cilostazol (10 μM), and 175.3 ± 23.9% (*P* < 0.001) by resveratrol (20 μM) in N2a cells (Fig 7B). The results suggest a mechanism for the neuroprotective effect of cilostazol against Aβ-induced neurotoxicity, that is, cilostazol-stimulated SIRT1 expression induces the phosphorylation and deacetylation of LKB1 (S428), which in turn leads to P-AMPK (Thr 172) and the inhibition of mTOR/P-P70S6K. Cilostazol upregulates autophagy by activating SIRT1/LKB1/AMPK1α signal pathways, and consequently deplete the intracellular Aβ and APP-CTFβ accumulation, and ameliorates neurotoxicity (Fig 7C).

## Discussion

In the present study, we demonstrate that cilostazol upregulates autophagy by activating SIRT1-coupled P-LKB1/P-AMPKα and inhibiting mTOR activation, thereby decreasing Aβ and APP-CTFβ accumulation. Cilostazol has shown neuroprotective effects against focal cerebral ischemia and chronic cerebral hypoperfusion injury by increasing cyclic AMP levels by inhibiting type III phosphodiesterase [18,19,29,30]. Recently, several reports have shown cilostazol is effective in ameliorating cognitive decline in patients with AD and cerebrovascular diseases [31] and mild cognitive impairment [32]. Most recently, it was emphasized that cognitive function can be maintained, even in a heterogeneous population with mild dementia by using cilostazol, which affects cerebral circulation and Aβ metabolism [21]. All together, these results suggest the beneficial effect of cilostazol in patients with AD.

Our previous study has shown that cilostazol stimulates autophagy by upregulation of beclin-1, Atg5, and LC3-II expressions through SIRT1 activation, thereby leading to depletion of intracellular Aβ [24]. However, the underlying mechanisms whereby cilostazol modulates the autophagic degradation of Aβ in neurons remain undefined. In the present study, P-ACC (a primary target of activated AMPK) significantly increased in parallel with P-AMPKα after exposing N2a cells to cilostazol. These effects of cilostazol were found to be comparable to the effects of resveratrol (SIRT1 activator) and AICAR (AMPK activator) on P-AMPKα and its downstream target P-ACC. Consistent with a report [33], resveratrol (20 μM) significantly increased the expression of P-AMPKα and of its downstream target P-ACC in N2a cells. This notion is further supported by the findings that pharmacological inhibitors; KT5720 (cAMP-dependent protein kinase inhibitor), compound C (a chemical inhibitor of AMPK), or sirtinol (SIRT1 inhibitor) blocked cilostazol (10 μM)-stimulated increases in P-AMPKα and P-ACC. These findings are well consistent with the report of Wu et al. [34], in that AMPK-SIRT1-autophagy pathway plays an important role in the neuroprotection by resveratrol. These results indicate that cilostazol exhibits a close relation with the activations of SIRT1 and AMPK.

Most intriguingly, the cilostazol-stimulated expressions of P-LKB1 and P-AMPKα exhibited SIRT1-dependency. AMPK is a master regulator of cellular energy homeostasis, a central player in glucose and lipid metabolism, and is potentially implicated in the pathogenesis of AD, for example, AMPK decreases in AD brains in association with decreased mitochondrial biogenesis [35]. Thus, pharmacological activation of AMPK by cilostazol shows a potential therapeutic target for ameliorating disrupted brain function induced by Aβ accumulation.

In addition, LC3-II levels were significantly elevated by cilostazol (10, 30 μM), indicative of enhanced autophagosome formation. It is known p62, a selective substrate for autophagy, is degraded by autophagy-lysosome pathway, and lysosomal inhibition results in marked accumulation of p62, indicating the p62 degradation by autophagy through direct interaction with LC3-II in lysosomes [25,36]. Further, cysteine protease cathepsin B was demonstrated to reduce Aβ peptide levels, especially the aggregation-prone species Aβ 1–42, through proteolytic cleavage [37]. Our results showed p62 expression was significantly decreased by cilostazol (10, 30 μM), whereas lysosomal enzyme cathepsin B activity was increased by cilostazol. Considering the report that p62 is degraded by autophagy through direct interaction with LC3 [25], it is likely that increased expression of LC3-II and lysosomal activation by cilostazol have resulted in increased p62 degradation.

To confirm that cilostazol-stimulated elevations of P-LKB1 and its downstream target P-AMPKα are mediated via SIRT1 activation, N2a cells were transfected with SIRT1 siRNA. After SIRT1 gene silencing, cilostazol failed to increase the expressions of P-LKB1 or P-AMPKα, whereas it obviously did so in cells transfected with scrambled siRNA duplex. These results indicate that increases in P-LKB1 and P-AMPKα were dependent on SIRT1 activation, and this speculation was further confirmed by the observations: when N2a cells were transfected with expression vectors encoding pcDNA SIRT1, increased P-AMPKα expression mimicked the effect of cilostazol, whereas cells transfected with mock pcDNA-transfected cells did not. These observations suggest cilostazol-stimulated expressions of P-LKB1 and P-AMPKα are mediated via SIRT1 activation. The functional relevance of LKB1 to AMPK signaling was further characterized by comparing the expressions of cilostazol- and resveratrol-stimulated P-LKB1 and P-AMPKα in N2a cells expressing LKB1 and in HeLa cells that do not LKB1 [38]. In HeLa cells, P-LKB1 and P-AMPKα were not detected prior to or after cilostazol or resveratrol treatment, indicating that cilostazol and resveratrol-stimulated expressions of P-AMPKα are LKB1-dependent.

In addition, mTOR has been reported to be a negative regulator of autophagy [6]. It was also reported that SIRT1 deficiency results in elevated mTOR signaling and that SIRT1 negatively regulates mTOR signaling potentially through TSC1/2 complex [17]. In the present study, expressions of P-mTOR and P-P70S6K were found to be significantly suppressed by cilostazol. Furthermore, consistent with the above-mentioned reports, cilostazol-stimulated expressions of P-LKB1 and P-AMPKα were mediated via SIRT1 activation. These findings were further confirmed by immunofluorescence study.

Based on these results and those of the pharmacological inhibition and gene silencing studies, it is concluded cilostazol strongly decreases intracellular Aβ peptide and APP-CTFβ accumulation by upregulation of autophagy through activating SIRT1-coupled P-LKB1/P-AMPKα, and thus ameliorates amyloid β-associated neurotoxicity.

## Materials and Methods

### Cell culture

Mouse neuroblastoma N2a wild-type cells and N2a cells stably expressing human APP Swedish mutation (N2aSwe cells) were kindly provided by Dr. Takeshi Iwatsubo (Department of Neuropathology and Neuroscience, Graduate School of Pharmaceutical Sciences, The University of Tokyo) [39]. When endogenous Aβ overproduction was required, N2a and N2aSwe cells were cultured under the above conditions in the presence of 1 μg/ml of tetracycline (Tet^**+**^, used as a control) for 48 hr, and then placed in medium deprived of tetracycline (Tet^**-**^), and cultured for 3, 12, or 24 hr. When treatment with cilostazol or resveratrol was required, cells were pretreated for 3 hr in Tet^+^ medium, and then switched to Tet^-^ medium and cultured for the indicated times.

### Western blotting

Cells were scraped and lysed using RIPA buffer (Sigma, St. Louis, MO). For Western blot analyses, proteins (30 μg) were loaded onto 10~15% SDS-polyacrylamide electrophoresis gels, electrophoresed, and transferred to nitrocellulose membranes (Amersham Biosciences, Inc., Piscataway, NJ), which were then incubated with anti-AMPKα anti-P-AMPKα (Thr 172), anti-acetyl-CoA carboxylase (ACC), anti-P-ACC (Ser 79), anti-SIRT1, anti-LKB1, anti-P-LKB1 (Ser 428), anti-P70S6K, anti-P-P70S6K (Thr 389), anti-mTOR, anti-P-mTOR, anti-p62/SQSTM1, anti-LC3A/B. Immunoblots were visualized by chemiluminescence using the Supersignal West Dura Extended Duration Substrate Kit (Pierce Chemical, Rockford, IL). Signals from bands were quantified using a GS-710 calibrated imaging densitometer (Bio-Rad, Hercules, CA).

### Small interfering RNA preparation and transfection

SIRT1 siRNA oligonucleotide (GenBank accession No. NM_003120.1) was synthesized by Bioneer (Daejeon, Korea). siRNA molecules were transfected into cells using X-tremeGENE siRNA transfection reagent (Roche, Indianapolis, IN), according to the manufacturer's instructions. siRNA sequences against SIRT1 were; ACGAUGACAGAACGUCACA (sense), and UGUGACGUUCUGUCAUCGU (antisense).

### SIRT1 overexpression experiments

Plasmids for wild-type SIRT1 (WT, pcDNA-Myc-His-SIRT1-WT) and empty pcDNA3.1 (mock control) were kindly provided by Dr. Jong Wan Park (Ischemic/Hypoxic Disease Institute, Seoul National University College of Medicine, Seoul). Cells were transfected with TransFast™ Transfection Reagent (Promega Corp., Madison, WI) according to the manufacturer's instructions. The transfection efficiency was confirmed by Western blot.

### SIRT1 deacetylation assay

SIRT1 deacetylase assays were performed using a fluorometric SIRT1 Assay Kit (Sigma). Briefly, the reaction was carried out at 37°C for 30 min. Deacetylase activity was detected as a fluorescent emission at 450 nm, with an excitation wavelength of 360 nm. The fluorescence intensity of the compounds at 450 nm was subtracted from the baseline values measured in the assay.

### Measurement of cathepsin B activity

To assess cathepsin B activity, cells were incubated in the presence of a fluorogenic cathepsin B substrate (100 μM, 219392, Enzo life sciences inc. Farmingdale, NY) for 1 h at 37°C. Fluorometry was carried out by measuring at 590 nm using a Tecan GeNios Plus (Tecan Group Ltd., Männedorf, Switzerland).

### Measurement of Aβ levels by ELISA

Cell lysates from cilostazol-treated and untreated cells were collected, and Aβ1–42 levels were determined using ELISA kit Aβ1–42 (FIVEphoton Biochemicals, San Diego, CA). Optical densities were read at 450 nm using a plate reader, and Aβ1–42 concentrations were determined using standard curves. All readings taken fell within the linear range of the assay.

### Immunocytochemistry

Cells were cultured in cover glass-bottomed dishes, fixed with 4% paraformaldehyde, permeabilized with 0.2% Triton X-100 in PBS. Expression of P-LKB or LC3-II or CTFβ were detected using anti-P-LKB or anti-LC3-II or anti-CTFβ antibodies. Cells were incubated with primary antibodies for 4 h, and then with Cy3- or FITC-conjugated secondary antibodies for 1 h. Fluorescent images were obtained using a confocal microscope (OLYMPUS FV-1000, Tokyo).

### Reagents and antibodies

Cilostazol [OPC-13013, 6-[4-(1-cyclohexyl-1*H*-tetrazol-5-yl) butoxy]-3,4-dihydro-2-(1*H*)-quinolinone] was donated by Otsuka Pharmaceutical Co., Ltd. (Tokushima, Japan), dissolved in 1% NH_4_OH to prepare a 10 mM stock solution, and diluted in DMSO (vehicle, <0.1% v/v of final volume). AICAR (5-aminoimidazole-4-carboxamide-1-β-D-ribofuranoside, an AMPK activator), resveratrol, and Compound C were from Sigma-Aldrich. Sirtinol (Calbiochem) was dissolved in DMSO. Aβ1–42 peptide was purchased from AnaSpec (AnaSpec, Fremont, CA). Anti-SIRT1 antibody from Santa Cruz Biotechnology Inc. (Santa Cruz, CA) and anti-CTFβ antibody from Calbiochem (La Jolla C). Anti-AMPKα, anti-P-AMPKα (Thr 172), anti-acetyl-CoA carboxylase (ACC), anti-P-ACC (Ser 79), anti-LKB1, anti-P-LKB1 (Ser 428), anti-P70S6K, anti-P-P70S6K (Thr 389), anti- p62/SQSTM1, anti-mTOR, anti-P-mTOR and anti-LC3A/B antibodies were purchased from Cell Signaling Technology (Danvers, MA).

### Statistical Analyses

Results are expressed as means ± SDs. One-way analysis of variance followed by Tukey’s *post hoc* multiple comparisons was used to determine the significances of differences between vehicle and cilostazol-treated groups. The Student’s *t*-test was used to determine the significances of differences between the means of untreated cells and those treated with inhibitors. Statistical significance was accepted for *P* values of < 0.05.

## Abbreviations

AD: Alzheimer disease
Aβ: amyloid-β
ACC: acetyl-CoA carboxylase
AICAR: 5-aminoimidazole-4-carboxamide-1-β-D-ribofuranoside
AMPK: AMP-activated protein kinase
APP-CTFβ: C-terminal APP fragment β subunit
CC: compound C
CR: calorie restriction
LKB1: liver kinase B1
N2a Swe cells: human APP Swedish mutation
mTOR: mammalian target of rapamycin
